# A New Prophylaxis and Cure for Gonorrheal Ophthalmia

**Published:** 1905-06

**Authors:** William Linton Phillips

**Affiliations:** Buffalo, N.Y.


469 Franklin Street.

---

### A New Prophylaxis and Cure for Gonorrheal Ophthalmia.

### With Special Reference to the New Born.

By WILLIAM LINTON PHILLIPS, M. D., Buffalo, N. Y.

FROM the beginning, man has run the gauntlet of the gonococcus while passing through the parturient canal. Even today, the offspring is subjected to this great danger and will be as long as one-sixth to one-third of the adult female, and three-quarters of the adult male population has this disease.

Even if we cannot wipe out the origin of gonorrheal ophthalmia we, nevertheless, may assure more or less immunity against it. Crède reduces the chances of infection from 10.8 per cent. to 0.2 per cent., and it becomes our duty entirely to eradicate it, if this be possible. Because 20 per cent. of the blind have been made so from lack of proper treatment at birth and that there still remains a danger of contraction of gonorrheal ophthalmia, I think it my duty once more to bring up a subject which has been written threadbare for, if there is any treatment that will reduce the chances of infection still further, it should be made known to the obstetrician, who sees these cases earlier than the ophthalmologist.

Symptoms of gonorrheal ophthalmia have been described and familiarised by nearly every one since time immemorial, but, nevertheless, will bear reviewing here, as it may reimpress some important point which has been forgotten and by so doing, save an eye. We may describe this disease under two general heads, antepartum and postpartum; but, because they differ only in their onset, they will be referred to in this paper under the one general head.

The rarity of the antepartum form is usually considered a blessing, but it is in reality a misfortune, for, if the maternal inoculation is not too long before birth, there is time enough to save the eye, while in the postpartum form many eyes are lost, due to the lack of diagnosis, owing to the discharge of the accoucheur before it develops. This, of course, is in the poorer class of patients.

Ophthalmia neonatorum usually develops in from three to four days after birth. Should it make its appearance later, say up to ten days, it is produced a great many times by germs other than the gonococcus, as Klebs-Loeffler bacillus, small bacillus of acute contagious conjunctivitis or the pneumobacillus. Often it comes from soiled towels, napkins or direct contact of the nurse's or mother's hands, when not properly cleansed.

The pathology presents a general engorgement, involving the vessels of the bulbar and palpebral conjunctiva; the superficial layer is infiltrated with leukocytes, in or on which the pathogenic microörganisms are seen. The conjunctival epithelial layer is swollen and uneven, caused by a serous or sometimes a fibrinous exudate. In the secretion from the eyes the gonococci are found free or in the pus cells.

Usually, in from twenty-four to thirty-six hours, there is a slight tendency for the eyelids to adhere, becoming puffed, and, as the disease approaches the third day, the eye becomes more tender to the touch; they, now, cannot be opened voluntarily. There is often a slight amount of fever and some swelling of the preauricular glands which lasts about two days. About the third day, the conjunctivæ are congested and swollen, but smooth. At this time the secretion develops, which is a mixture of blood and serum and has been described as resembling bouillon.

At the fourth or fifth day, the eyelids begin to thicken and appear dark red in color. The secretion now assumes a more purulent form, which may be thin enough to flow out of the eyes freely, or else so thick that it is retained, welling out when the eyelids are separated. Later on, the swelling begins to disappear. The skin now assumes a more wrinkled appearance, the eyes are also less tender to the touch and the conjunctivæ gathered in soft folds.

In the second stage, which may persist for weeks, the cornea is in the greatest danger, owing to the retention of pus within the eyelids, which now has a more constant action, producing perforated cornea, staphyloma, ulcers, vascularisation, pyramidal cataracts, and central macula, depending upon the severity of the disease, whether contracted from gleet or a virulent gonorrhea.

In its severe form there is a great many times a deposited membrane upon the conjunctiva, having the appearance of being croupous or diphtheritic in character.

Because modern science teaches that cleanliness is the most essential step in the prevention of disease, this paper deals with prophylaxis rather than with the cure of gonorrheal conjunctivitis after it develops.

To understand fully the prevention of this disease, we must consider, first, the origin from which it springs; and know, second, along which paths it may travel. The former is a fact well known, but the latter is more difficult to understand, unless we look over a few truths in relation to childbirth. When the fetus starts downward on its course, the eyes no doubt are uninfected because of the protection afforded by the amniotic sac; during the descent they become exposed to the infection, caused by the rupture of the sac and the forcing apart of the eyelids, produced by the dragging of the vaginal membranes upon the face. The mouth at this time may also be forced open from the resistance offered the lower jaw.

The greatest percentage of all infected eyes which occur at birth are due to the direct contact with the gonococcus. The minimum amount travels indirectly by other paths and reaches the eye through the nose, a cavity which, with but few exceptions, remains open, becoming filled with septic material which is drawn still further back by the first breath filling all openings.

We may consider two ways by which infection travels to the eye from the nose; first, by the bloodvessels and lymphatics; second, the tear ducts. Because the latter is the more direct, we will speak of it as the only way, although bacteria have been traced through the vascular system. It has been proven by numerous cases on record, that air may be forced through the tear ducts to the eye and into the surrounding tissues of the orbit by blowing, sneezing or coughing. How easy may the gonococcus be forced the same way by the act of sneezing, which nearly always follows the exposure of the child to different temperatures at birth.

It is a simple thing to prove that free drainage through the tear ducts will be beneficial in phlyectenular conjunctivitis, ulcers, and the like, because of the removal of irritating fluids; but it is quite another task to prove to the skeptical mind that gonorrheal ophthalmia in the newborn does spread by way of this route, although this paper has already proven this theoretically.

In August, 1899, I was consulted about Miss L. Z., who at this time was suffering with erysipelas of the upper lip, beneath the nostril. The initial spot was about 10 mms. in diameter. Her family physician informed me she had suffered with this disease before, but not in this particular way. From the point of infection it spread rapidly by way of the mucous membrane of the nose to tear ducts which were greatly swollen, inflamed and filled with pus, showing clearly the way it traveled. The disease did not spread to any part of the face, except as mentioned, which point was first cured. Later, the nose responded to treatment and last, the eyes.

Since this case my attention has been called to this route of infection many times, but none so forcibly as the following:

Mr. R:, 17 years old, suffered all the previous summer with catarrh of the right nostril, as he described it; the corresponding eye was slightly involved, appearing red and painful at times, later these symptoms became aggravated and more persistent, photophobia developed, and on November, 1903, he consulted a physician, who gave treatment daily for three months, without relief. On February 9, 1904, I found Mr. R. suffering with an iritis; the cornea very cloudy; conjunctiva, both palpebral and bulbar, deeply engorged and the iris firmly bound down. A nasal examination showed turbinates greatly swollen, deeply injected and painful to the touch; on the septum was a large ulcer; there was also constant running of the right nostril, the left appeared normal. Daily applications were made to the nose, and in three weeks the patient was cured. The eye showed improvement only in proportion to that of the nose and did not respond to medicines dropped in the eye.

These cases ought to show, conclusively, without reporting any more, that the nose, in a great many cases, is the true origin of diseases of the eye.

Installation of silver nitrate in the eyes of the newborn seems universally conceded sufficient protection against gonorrheal infection, but is shown by this still remaining 0.2 per cent. that it is insufficient. This, then, should prove convincing enough to the most skeptical mind that obstetricians should carry on a strict nasal hygiene for its prevention. But how is this to be accomplished? First, there must be found a suitable drug that will overcome the following difficulties: It must be nontoxic, for we are dealing with a most delicate system which may become poisoned. It also must not coagulate albumins as bichloride of mercury, carbolic acid, copper sulphate or silver nitrate, for this has a tendency to afford a protection to the gonococcus rather than destroy it. It also must be nonirritating, no matter in what strength used; and, lastly, it must be powerful enough to destroy germs.

The most admirable drug, embracing all these most important features, is alphozone or succinic peroxide. Not only is it nontoxic, nonirritating, nonpainful and does not coagulate albumins, but it is powerful enough to kill the colon bacillus, pyogenic and pathogenic germs in one minute, if placed in a 1 to 250 solution. Alphozone is 100 times more powerful than hydrogen peroxid and does not produce effervescence, which would spread the infection throughout the surrounding tissue. Alphozone is 75 times more powerful than carbolic acid, with its many disagreeable properties missing. It is 6 times more germicidal than silver nitrate and in the same proportions is equal to bichloride of mercury solutions, but superior to it in many ways, as previously stated.

Last summer an opportunity presented itself for using alphozone, while in consultation with another physician, who was using large doses of protargol for one week to check a virulent ophthalmic gonorrhea with no result. It was ordered by my advice, pushed to its physiological limits, but with no better result. Argyrol and other drugs ended likewise, so, as a last resort and in conjunction with protargol, alphozone was prescribed. A 1-500 solution was dropped in the eyes hourly. The next day the diphtheritic-like deposit and the pus began to disappear. Upon the second day, under my care, protargol was stopped and the tenth day the patient was discharged without any complications. I have since used this drug alone and more extensive in septic conditions of the tear ducts, eyes and in all operations and found it prevented all septic conditions.

Not long ago, I was called in to see a case which developed ten days after birth; silver nitrate had been used hourly for one week with no result; upon separating the lids three-quarters of a teaspoonful of greenish yellow pus flowed out of the eye; after cleansing, it was seen that the cornea had ruptured through an ulcer which was about 10 mms. in diameter. A 1-30 solution of alphozone was ordered dropped in the eye, hourly, night and day. All other medications were stopped except atropine sulphate, which was used in dilating the pupil. The nostrils were cleansed with this same solution. Twenty-four hours from the time of the commencement of this treatment there remained in the eye, near the canaliculus, one small drop of pus; the cornea had healed, leaving no involvement of the deeper structures. By the fourth day the pus entirely disappeared and did not return. The case was discharged at the end of the fifteenth day, leaving no complications.

It might be well to mention that alphozone oxidises sharp steel instruments and by so doing dulls them, if left in solution for more than a minute. New solutions are not so powerful as those having been made twenty-four hours and alphozone may be kept in solution indefinitely if well corked.

Having found the most suitable drug, filling all these requirements, it is not hard to find a way to administer it, although great care must be taken while so doing, in order not to force the septic material further back into the Eustachian tubes. Because of their low position in the throat of the child, the head should be tipped forward so that the fluid will flow out of the nose again rather than into the throat for the same reason.

Alphozone may be administered by a medicine dropper if a very gentle pressure is applied to the bulb, but the safest way is to spray it with an atomiser, thoroughly. Alphozone dissolves readily in water at ordinary temperature and the action is not impaired providing the water does not over-reach the boiling point at the time of making the solution. It forms an odorless, white, painless solution, which may be used in any degree of strength from 1 in 30 upwards, although I have found in operations and diseases of the eye that solutions 1 to 250 of water, answers all practical purposes, but at times stronger solutions are necessary.

The nose should be cleansed several times a day, if certain that there was a gonorrheal discharge before birth; if not, one good spraying will do. The mouth should also receive like care and the tear ducts gently massaged towards the canaliculus to force out the remaining pus, after which alphozone 1 in 30 should be freely dropped in the eye. In recommending alphozone to replace Créde's method, I think that a solution 1 in 30 or weaker, dropped in the eyes freely at birth, will prove advantageous over silver nitrate, if but for the facts that it does not coagulate albumens, allowing deeper penetration into the conjunctival folds, also tear ducts, and because stronger solutions may be used freely without danger of a drug, which is six times more powerful than silver nitrate in equal proportions.

449 FRANKLIN STREET.

